# Ectopic Pregnancy in a Non-communicating Rudimentary Uterine Horn: A Case of Successful Medical Management and Literature Review

**DOI:** 10.7759/cureus.71856

**Published:** 2024-10-19

**Authors:** Amelie M Harpey, Bridget P McNierney, Alexis A O'Connell, Emily G Lingo, Ellen G Wood

**Affiliations:** 1 Obstetrics and Gynecology, Philadelphia College of Osteopathic Medicine, Philadelphia, USA; 2 Obstetrics and Gynecology, Hospital Corporation of America (HCA) East Florida, Fort Lauderdale, USA; 3 Reproductive Endocrinology and Infertility, IVFMD-South Florida Institute for Reproductive Medicine, Cooper City, USA

**Keywords:** cornual ectopic pregnancy, infertility, mullerian uterine anomaly, rudimentary horn, unicornuate uterus with distal non-communicating uterine horn

## Abstract

A non-communicating rudimentary uterine horn is a Müllerian abnormality that manifests due to abnormal Müllerian duct development. This abnormality is associated with endometriosis, infertility, and pregnancy complications, including ectopic pregnancy, abnormal fetal presentation, abruption, increased fetal mortality and morbidity, preterm rupture of membranes, preterm birth, intrauterine growth restriction, and uterine rupture. If pregnancy does occur, there is a high risk of complications, most notably rupture of the rudimentary horn. For this reason, such pregnancies are managed similarly to an ectopic pregnancy with either administration of methotrexate, laparoscopic removal, or a combination of both. This article describes a case of a pregnancy in a previously diagnosed, non-communicating rudimentary uterine horn that was managed medically. We aim to explore the need for early identification of rudimentary uterine horns and the management of pregnancy in these individuals.

## Introduction

During embryonic development, the Müllerian ducts evolve to form the uterus, the fallopian tubes, and the upper two-thirds of the vagina. As they develop, the Müllerian ducts undergo elongation, cannulation, fusion, and resorption of the septum. Disruptions in any stage of this process can result in Müllerian abnormalities such as unicornuate uterus, didelphic uterus, bicornuate uterus, uterine septum, and arcuate uterus [1].

Müllerian duct abnormalities are often associated with infertility. The prevalence of congenital uterine anomalies is 3.5% of infertile women, compared to 0.17% of fertile women [2]. This suggests that uterine anomalies are 21 times more prevalent among infertile women [3]. Several obstetric complications, including fetal malpresentation, preterm delivery, and fetal growth restriction, can occur once a patient becomes pregnant. These complications may be in part due to abnormal uterine vascular supply and decreased myometrial mass [4].

The presence of a rudimentary horn may be referred to as either communicating or non-communicating, describing whether the horn has an anatomical opening to the uterine cavity. Some individuals have functional endometrial tissue within this rudimentary horn, and these patients may have an increased risk of ectopic pregnancy, endometriosis, abnormal fetal presentation, fetal growth restriction, and preterm delivery. A review of pregnancy outcomes conducted by the American Society of Reproductive Medicine (ASRM) found an ectopic pregnancy rate of 2.7%, compared to 2% in a uterus without anomalies [1]. If a rudimentary horn pregnancy (RHP) is diagnosed, excision of the pregnant horn is recommended because of the high risk of rupture of an RHP in the second trimester [2].

Making the diagnosis of a unicornuate uterus requires imaging. Hysterosalpingogram (HSG) is one of the modalities used to diagnose various Müllerian anomalies. A unicornuate uterus will show characteristic findings of unilateral opacification of the fallopian tube with a uterus that is shifted toward the ipsilateral side. Ultrasound with optional magnetic resonance imaging (MRI) confirmation is best for diagnosing a rudimentary horn, especially if the horn(s) are non-communicating. 3D ultrasound increases diagnostic accuracy by obtaining reconstructed images in the coronal plane, which depicts the unicornuate uterus's deviation and the endometrium's characteristic appearance [4]. Such imaging also allows us to confirm whether or not the endometrium is present and functional and if there is communication with the central uterine cavity. This is important to know, especially in the setting of ectopic pregnancy, which predisposes women to devastating consequences such as uterine rupture and life-threatening bleeding [4].

Our case describes the diagnosis and management of a woman with a known unicornuate uterine anomaly and implantation of a pregnancy in the rudimentary uterine horn.

## Case presentation

**Figure 1:**
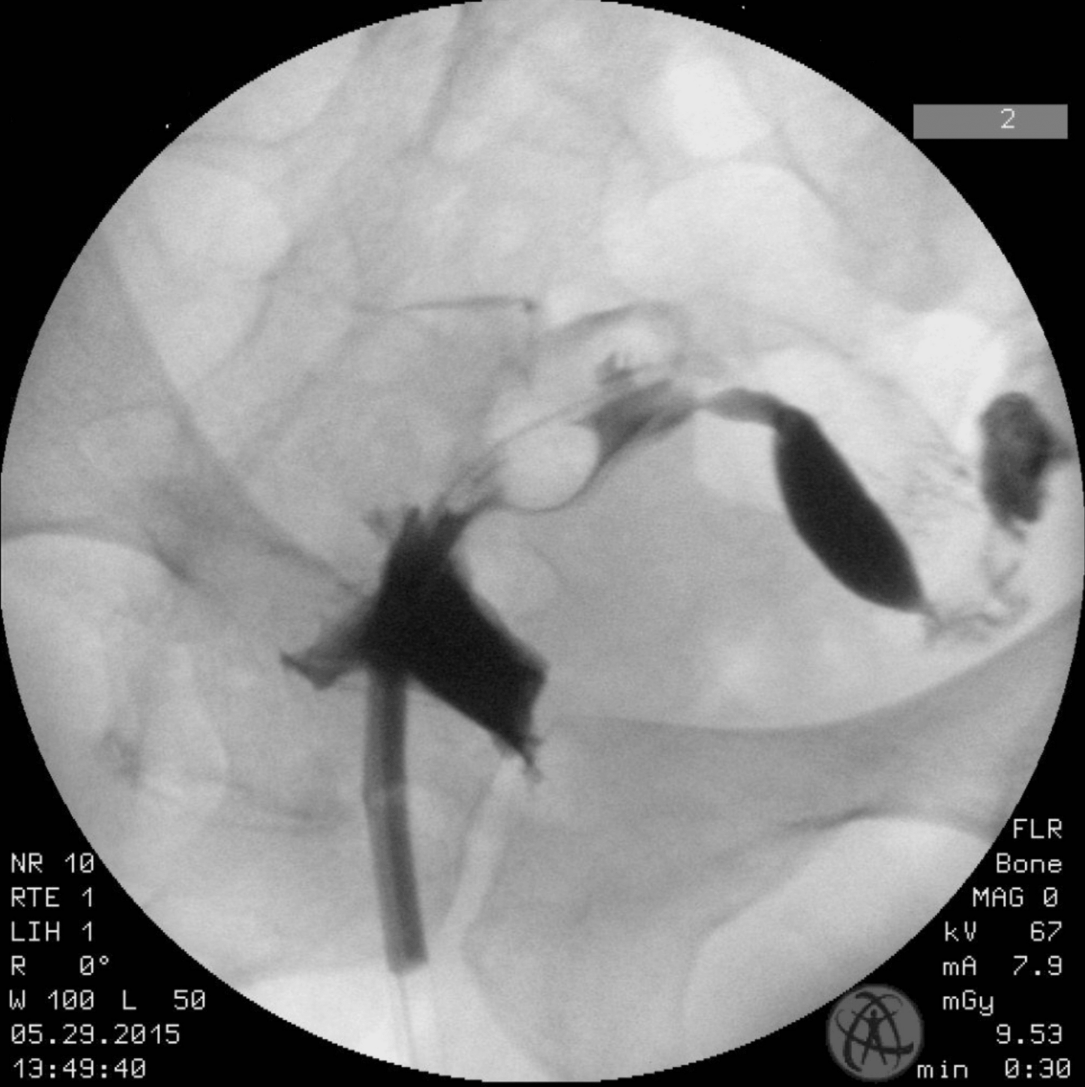
A hysterosalpingogram showing a left-sided hemiuterus with a patent left fallopian tube and a typical left-sided spill pattern, with the absence of the right side of the uterus and right fallopian tube.

A 37-year-old nulligravid female presented to the infertility clinic for an initial consultation regarding her fertility potential. During her initial evaluation, a transvaginal ultrasound (TVUS) identified a uterus with a potential uterine anomaly. The patient was scheduled for HSG to assess the patency of her fallopian tubes and her uterine contour. The study showed a left-sided unicornuate uterus and patent left fallopian tube, with no dye extravasation into the right side of the uterus and no tubal patency on the right side. A pelvic MRI was ordered to confirm the diagnosis, and the report stated "unremarkable pelvic MRI with and without gadolinium" and noted a "dominant 15mm right ovarian follicular cyst." However, clinical suspicion for a uterine anomaly remained high given her initial visit in-office ultrasound and HSG findings. A subsequent intravenous pyelogram was ordered, which revealed bilateral kidney function. The diagnosis of a left unicornuate uterus with a possible right uterine horn (Figure 1) was made. 

**Figure 2:**
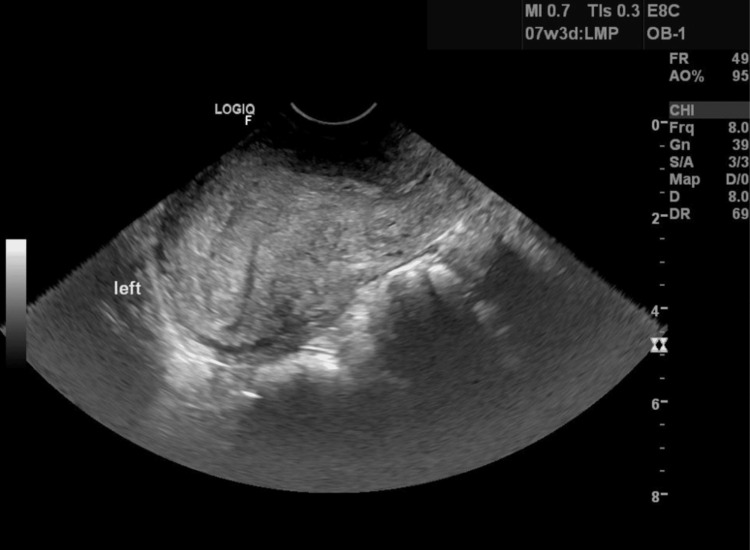
A transvaginal ultrasound of the intrauterine cavity at the time of diagnosis of the pregnancy within the rudimentary uterine horn.

**Figure 3:**
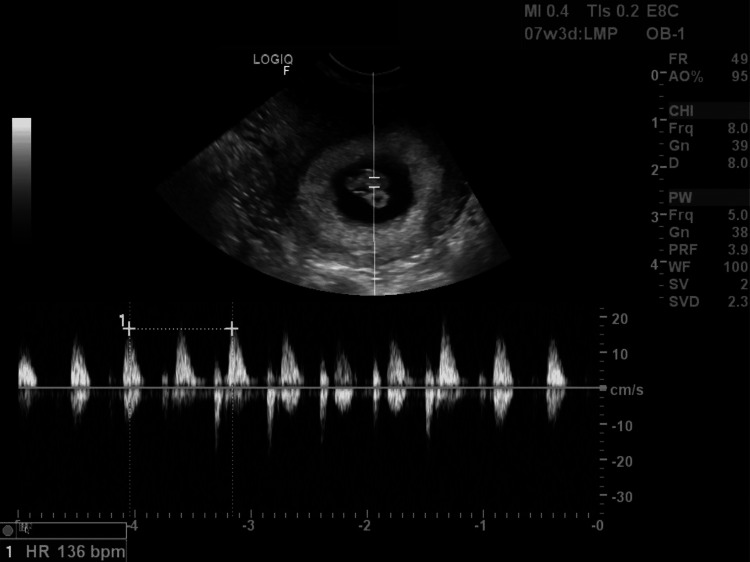
A transvaginal ultrasound of the fetus with present cardiac activity at 7w3d in what is suspected to be a right rudimentary uterine horn.

Five months later, the patient returned to the office with an unplanned singleton intrauterine pregnancy. Unfortunately, the pregnancy ended in a spontaneous abortion. A year later, the patient presented again with a positive home pregnancy test. TVUS showed an empty intrauterine cavity (Figure 2) and a pregnancy that was suspected to be in a non-communicating rudimentary right horn. The pregnancy measured seven weeks and three days, with a corpus luteal cyst noted on the right ovary (Figure 3). Her beta-human chorionic gonadotropin (hCG) levels at this time were 25,279 mIU/mL, with an estradiol (E2) level of 339 pg/mL and a progesterone (P4) level of 24.1 ng/mL. A second MRI of the pelvis was ordered to confirm an ectopic pregnancy, and the report stated, "There appears to be a small/early pregnancy in the right pelvis. Given the patient's history, this may be within a remnant right-sided endometrial horn. It cannot be clearly connected to the dominant left-sided endometrial horn canal from the patient's reported unicornuate uterus."

**Figure 4:**
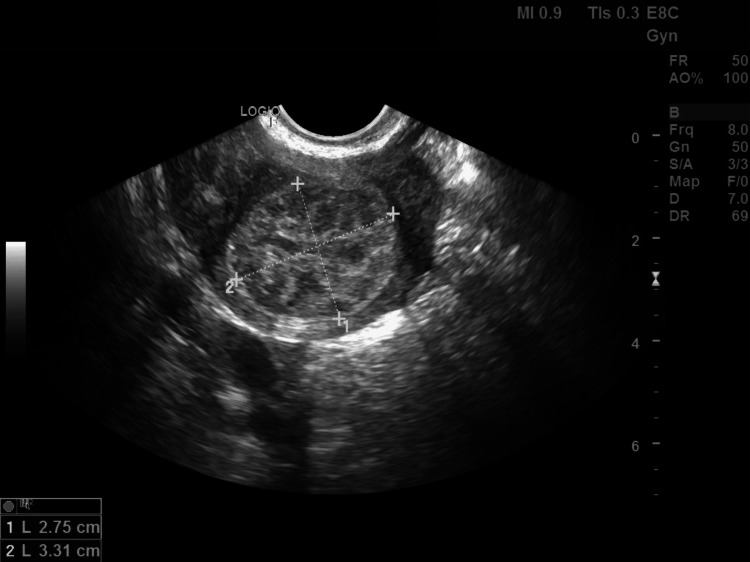
A transvaginal ultrasound of the pregnancy remnants measuring 2.75cm x 3.31cm once beta-hCG levels returned to undetectable levels. This image was obtained six months after administration of methotrexate for medical termination of pregnancy in the rudimentary uterine horn.

Based on this patient's earlier imaging, it was speculated that spontaneous ovulation occurred, and the sperm traveled via the left fallopian tube, fertilized an oocyte from the right ovary, and implanted in the endometrium of the rudimentary horn via the intra-abdominal cavity. Upon diagnosis of a rudimentary uterine horn ectopic pregnancy, the patient was referred to her OB/GYN and Maternal Fetal Medicine (MFM) providers. The MFM recommended treating the patient medically with methotrexate due to the diagnosis being made at an early gestational age. The patient received two doses of methotrexate, which terminated the heartbeat and the pregnancy. Five months after the methotrexate injections, the quantitative beta-hCG level was undetectable. At this time, follow-up imaging was performed; the sonogram showed persistent clot formation measuring 2.75 x 3.31 cm (Figure 4), which was consistent with the remnants of medical termination of pregnancy.

**Figure 5:**
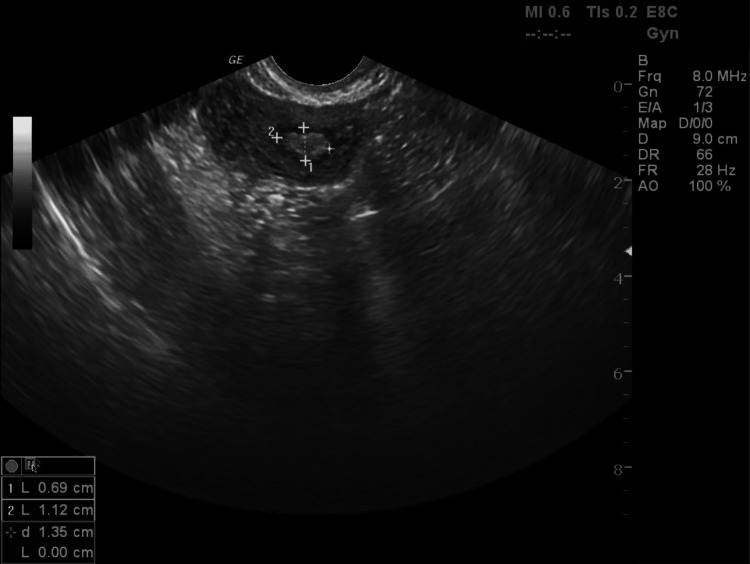
A transvaginal ultrasound of the pregnancy remnants in the rudimentary uterine horn, measuring 0.69 x 1.12cm 14 months following administration of methotrexate for medical termination of the pregnancy, at the time of second frozen embryo transfer.

Once the beta-hCG was negative, she returned to the infertility clinic to pursue fertility treatment. She subsequently had two biochemical pregnancies: one three months after a negative beta-hCG and the second five months later. She then decided to move forward with IVF. In her IVF cycle, she underwent a transvaginal oocyte retrieval yielding seven oocytes, which grew into three blastocysts that were biopsied for aneuploidy. Two euploid embryos were identified. The first embryo was successfully implanted, yielding a healthy full-term baby girl via C-section; however, the patient was placed on bed rest at 24 weeks due to the risk of preterm labor, likely due to her unicornuate uterus. This pregnancy was successful despite the presence of the clot remnants within her rudimentary uterine horn from her previous medical termination of pregnancy. The rudimentary uterine horn and ipsilateral fallopian tube were not removed at the time of delivery by the delivering obstetrician. The patient presented a year later for a second frozen embryo transfer (FET). At this time, an ultrasound was performed and showed that the clot remnants previously seen following her medical termination of pregnancy were down to 0.69 x 1.12 cm (Figure 5). The FET resulted in a successful implantation; however, the pregnancy ended as a missed abortion. The patient was not treated further by the reproductive endocrinologist.

## Discussion

In patients with a uterus that lacks Müllerian abnormalities, extrauterine pregnancies can be managed medically or surgically. Medical management of a tubal ectopic pregnancy is achieved with the use of methotrexate. This modality should be employed in hemodynamically stable patients whose pregnancy is unruptured and who do not have absolute contraindications to the use of methotrexate. Prior to the use of methotrexate, some clinicians may choose to induce fetal demise medically. Some of the reasons include fears of legal retaliation, providing emotional comfort to the patient and providers involved in the procedure, a belief that dilation and evacuation are easier and faster, avoiding transient fetal survival following medical induction, and avoiding extramural delivery with signs of life. The most commonly used medications include digoxin, potassium chloride (KCl), and lidocaine [5]. In the event of a second-trimester abortion, direct fetal intracardiac potassium chloride injection effectively causes immediate fetal cardiac arrest. This approach may be adopted to ensure that the abortus is stillborn [6]. This can also be performed for any nonviable pregnancy, regardless of the gestational age. Following induction of fetal demise, methotrexate can be administered according to one of three protocols. The single-dose protocol involves a single 50 mg/m^2^ intramuscular dose. The two-dose regimen is a 50 mg/m^2^ IM dose on day one, followed by a second dose on day four. The fixed multiple-dose regimen involves a weight-based 1 mg/kg IM dose administered on days 1, 3, 5, and 7, alternating with a 0.1 mg/kg dose of Leucovorin on days 2, 4, 6, and 8. Notable absolute contraindications include intrauterine pregnancy, immunodeficiency, hepatic or renal insufficiency, and those unable to follow up reliably [7]. After administration of methotrexate, the patient must be closely followed to ensure resolution of the ectopic pregnancy and monitor for signs of rupture. This is performed with serial beta-hCG and ultrasound monitoring. If beta-hCG levels do not decrease appropriately, surgical management must be considered.

Surgical management includes laparoscopic salpingectomy or laparoscopic salpingostomy; if the patient is unstable or if there is inadequate visualization, a laparotomy is performed. Surgical management can be performed in any patient who prefers it to medication. It is indicated if the patient is hemodynamically unstable, there is evidence of a ruptured ectopic, there are signs of intraperitoneal bleeding, the patient has an absolute contraindication for the use of methotrexate, or initial management with methotrexate fails [7]. 

A literature review of 73 cases in PubMed on ectopic pregnancies in rudimentary uterine horns was performed. Exclusion criteria included patients who had live deliveries despite pregnancy in a rudimentary uterine horn. Of the cases reviewed, only five were successfully treated medically. One case injected 100mg of methotrexate directly into the gestational sac, one involved intra-amniotic methotrexate, one received intraplacental methotrexate, and the other two administered a 50mg intramuscular dose of methotrexate. These five patients all underwent laparoscopic removal of the rudimentary uterine horn following medical management of the ectopic pregnancy. Of those 73 cases, 68 were treated surgically. 41 patients underwent a laparotomy, and 27 underwent laparoscopic resection. In four cases, fetal demise was induced prior to further management. Two cases used KCl, and two cases used intracardiac lidocaine. The average gestational age of the cases treated medically was approximately seven weeks of gestation. The gestational age for surgical management ranged from six weeks to 34 weeks. It appears that an ectopic diagnosed at an earlier gestational age is more likely to be treated medically. More advanced pregnancies carry a higher risk of complication if medical management fails, notably uterine rupture. Of the cases reviewed, 29 pregnancies were complicated by rupture of the rudimentary uterine horn. Here, we make note of some more complicated cases of ectopic pregnancies that required surgical management. 

While tubal ectopic pregnancies occur in over 95% of cases, they can also occur in the ovary, abdomen, cervix, broad ligament, and uterine horns. A case was presented in 2021 of a patient who underwent laparotomy for diagnosis of right cornual pregnancy with a secondary abdominal pregnancy at 29 weeks gestation, confirmed on ultrasound and MRI. During the procedure, they found that the placenta had been implanted on a right-sided uterine horn and determined that the fetus and placenta had entered the abdominal cavity following the rupture of the right uterine horn. They delivered a live male infant from the abdominal cavity without further complications to the mother. Abdominal pregnancy resulting from a ruptured uterine horn is extremely rare, and this case further emphasizes the importance of early detection and management of these ectopic pregnancies. A high index of suspicion, coupled with appropriate imaging studies, can help facilitate its diagnosis in any suspected case [8].

Failure to diagnose a uterine horn pregnancy can lead to rupture and internal hemorrhage [9], which can lead to further life-threatening complications. In another case report, a mother died secondary to rudimentary horn rupture following an undiagnosed ectopic pregnancy measuring 19 weeks gestation. The growth of the fetus into the second trimester was believed to have caused the rupture of the horn and led to hemorrhagic shock in the mother and ischemic death in the fetus. This report shows that ruptured ectopic pregnancies are a possible cause of unexpected death in fertile women and further underlines the importance of early detection of these Müllerian duct anomalies by sonography during routine obstetric examination. In 67% of cases, the rudimentary horn most frequently ruptures in the second trimester and is often associated with fetal demise. Additionally, any woman presenting with signs of acute abdomen, intraperitoneal hemorrhage, and shock should be evaluated for anomaly-related ectopic pregnancies should they be undiagnosed at that time [9]. 

As emphasized throughout this report, early diagnosis of ectopic pregnancies in rudimentary horns is crucial in optimizing morbidity and mortality in patients. A similar case to the one presented in this paper found that preoperative medical termination of a cornual ectopic with subsequent laparoscopic excision of the uterine horn provided the best results for the patient [10]. In this particular protocol, the patient received a 60mg dose of IM methotrexate and underwent a TVUS-guided puncture of the gestational sac with drainage of extraembryonic fluid and administration of 50mg of methotrexate into the sac. Her beta-hCG levels trended down appropriately, and her ultrasound showed progressive regression of the trophoblastic content. Three months later, the patient underwent laparoscopic resection of the right uterine horn with right salpingectomy to prevent additional cornual ectopic pregnancy. This particular patient's uterine horn had previously been misdiagnosed as a pedunculated myoma [10]. Incorrect diagnosis of these uterine anomalies is unfortunately prevalent, as appropriate imaging is not always readily available or can often be misread due to the amount of anatomic variation that can be seen in these Müllerian abnormalities [8]. While TVUS is the gold standard for the diagnosis of uterine and adnexal pathology, it has a low sensitivity for the diagnosis of a rudimentary horn. Such abnormalities are better seen and distinguished on MRI and 3D ultrasound [10]. 

If an ectopic pregnancy within a rudimentary horn is diagnosed within the first trimester, standard treatment can involve surgical excision of the horn and ipsilateral fallopian tube. Surgery is recommended, not only due to the high risk of rupture in the second trimester but also to prevent recurrence. However, asymptomatic and hemodynamically stable patients may undergo medical termination before surgery. This additional measure decreases blood flow to the horn, allowing for easier surgical management and reduced blood loss during surgery [10]. Most cases of successful laparoscopic resection of unruptured ectopic pregnancies in uterine horns have been done on patients with low beta-hCG levels with or without cardiac activity, but this is not a requirement for successful excision of the ectopic with minimal blood loss [11]. Determining the acuity of the patient and the risk of rupture can be used to determine if medical or surgical management is the most appropriate first line. Medical management is effective in terminating the pregnancy and preventing the risk of cornual rupture during the current pregnancy. Still, it is not a definitive treatment, and future surgical intervention should be considered [10]. 

Occasionally, there is suspicion of ectopic pregnancy without an initial diagnosis of Müllerian abnormality. Regardless of the cause of the ectopic pregnancy, it should be treated to prevent rupture. In the case of a 39-year-old nulligravid female with suspected left tubal ectopic pregnancy, a laparoscopy was performed for left salpingectomy and removal of the tubal ectopic pregnancy. Instead of seeing prominent tubal ectopic findings, a right unicornuate uterus with a dilated rudimentary horn was seen. The pregnancy was excised from the rudimentary horn, and a left salpingectomy was performed, with complete resolution of a left rudimentary horn ectopic pregnancy by surgical excision of the pregnancy without hemi-hysterectomy. The decision was made not to perform a hemi-hysterectomy due to the absence of patient consent regarding the excision of the rudimentary horn. The patient later underwent an MRI that confirmed her right unicornuate uterus with a non-communicating left rudimentary horn [12]. Being aware of Müllerian anomalies when diagnosing ectopic pregnancies may help determine the patient's course of treatment, as unexpected anomalies can impact standard operative management of an ectopic pregnancy [10]. 

Surgical resection of rudimentary horns is the most definitive treatment for cornual ectopic pregnancies. It is generally recommended to avoid further progression of the pregnancy, leading to cornual rupture and intraperitoneal hemorrhage. However, methotrexate can be an alternative or adjunct to surgery early on in gestation. There is a case of a 24-year-old female presenting to a clinic for an elective termination of pregnancy. She failed surgical abortion due to the persistence of the gestational sac and was sent to a tertiary center for further evaluation. Her office ultrasound revealed two uterine cavities with a pregnancy located in the right uterine horn. Entry into the right-sided cavity was attempted under ultrasonographic guidance to evacuate the pregnancy but was unsuccessful. At this time, the patient opted for medical management. The patient received a single dose of methotrexate and a dose of vaginal misoprostol. One week later, there was a loss of fetal cardiac activity, but the patient denied bleeding and cramping. She received a second dose of misoprostol with no changes in her presentation. The patient underwent serial ultrasound monitoring for three months, which showed continued degeneration of the pregnancy tissue with no evidence of expulsion. The patient remained asymptomatic. Six months following her elective abortion, the patient opted for diagnostic imaging to evaluate the effects of her anomaly on her future fertility and health. The MRI showed a right rudimentary horn, which was determined to be non-communicating, and revealed the presence of tissue that was continuing to degenerate. The patient elected for surgical removal of the horn, and the final pathologic diagnosis showed decidual changes with chorionic villi and decidua with marked degenerative changes [13]. 

In this case report, the patient initially opted for medical management of her cornual ectopic pregnancy. She received two doses of methotrexate in October, and it took until March for her beta-hCG levels to return to normal. She had serial ultrasounds to monitor the progression and degeneration of the pregnancy, and the clot and pregnancy remnants took over a year to fully resorb. Serial ultrasounds are essential when opting for medical management of ectopic pregnancies, as the US features of the endometrium, mainly endometrial thickness and echogenicity, are used to determine the need for surgical intervention. While endometrial thickness is a poor predictor of the need for surgical intervention, ultrasonography is an essential part of the examination, leading to the decision of secondary intervention of early medical abortions. This study in Denmark emphasized considerable variation in endometrial thickness and echogenicity and that as long as there was a pattern of change in these features over time, further surgical intervention was not needed [14]. This patient did not undergo further intervention despite the tissue taking over a year to degenerate and resorb, as her follow-up with regular ultrasound continued to show progression and change in the endometrium. That patient remained hemodynamically stable and asymptomatic and did not desire surgical intervention. There does not appear to be a timeframe in which pregnancy remnants should be evacuated and resorbed from a rudimentary horn, particularly non-communicating ones. There seems to be little data published at this point, as these types of ectopic pregnancies continue to be rare.

## Conclusions

Similar to patients with ectopic pregnancies and a uterus of normal morphology, patients with a pregnancy located in a rudimentary uterine horn can be managed medically or surgically; there is no standard protocol at this time to guide the management of these patients. Our patient underwent medical therapy as recommended by MFM, which resulted in a markedly prolonged period of resorption. Medical management can be used alone as an alternative to surgery, as an adjunct to surgery, or it can be foregone if the patient elects to manage the pregnancy with surgery alone. Even if medical management effectively terminates the pregnancy, surgery is the more definitive option to resolve the uterine anomaly and prevent issues with future pregnancies. The best course for these patients is to diagnose a rudimentary uterine horn early on before becoming pregnant, using imaging modalities like HSG, 3D ultrasound, and MRI, as they are the most accurate in revealing the true morphology of the abnormality. Furthermore, the development of a standardized protocol for managing rudimentary uterine horn pregnancies will help reduce future obstetric complications in these patients. 
